# Microsolvation and sp^2^-stereoinversion of monomeric α-(2,6-di-*tert*-butylphenyl)vinyllithium as measured by NMR

**DOI:** 10.3762/bjoc.10.263

**Published:** 2014-10-29

**Authors:** Rudolf Knorr, Monika Knittl, Eva C Rossmann

**Affiliations:** 1 Department Chemie, Ludwig-Maximilians-Universität München, Butenandtstrasse 5–13 (Haus F), 81377 München, Germany

**Keywords:** ^13^C,^6^Li NMR coupling, ion pair intermediate, monomeric alkenyllithiums, pseudoactivation parameters, sp^2^-stereoinversion mechanism, THF catalysis

## Abstract

The β-unsubstituted title compound dissolves in THF as a uniformly trisolvated monomer, whereas it forms exclusively disolvated monomers in *tert*-butyl methyl ether, Et_2_O, TMEDA, or toluene with TMEDA (1.4 equiv). This was established at low temperatures through the observation of separated NMR signals for free and lithium-coordinated ligands and/or through the patterns and magnitudes of ^13^C,^6^Li NMR coupling constants. An aggregated form was observed only with Et_2_O (2 equiv) in toluene as the solvent. The olefinic geminal interproton coupling constants of the H_2_C= part can be used as a secondary criterion to differentiate between these differently solvated ground-states (3, 2, or <2 coordinated ligands per Li). Due to a kinetic trisolvation privilege of THF, the cis/trans sp^2^-stereoinversion rates could be measured through analyses of ^1^H NMR line broadening and coalescence only in THF as the solvent: The pseudomonomolecular (because THF-catalyzed), ionic mechanism is initialized by a C–Li bond heterolysis with the transient immobilization of one additional THF ligand, followed by stereoinversion of the quasi-sp^2^-hybridized carbanionic center in cooperation with a “conducted tour” migration of Li^+^(THF)_4_ along the α-aryl group within the solvent-separated ion pair.

## Introduction

**Scheme 1:**
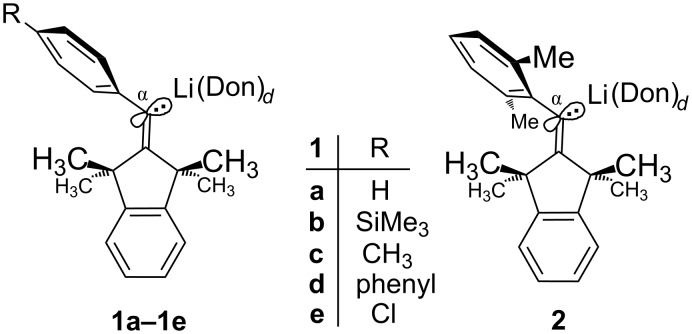
The sterically congested model systems.

Organolithium compounds tend to aggregate in solution unless the structure of their carbanionic part favors the nonaggregated (monomeric) species [2]. For example, tetrameric and dimeric *n*-butyllithium (*n-*BuLi) can coexist [3] in a highly mobile equilibrium, so that their reliable kinetic differentiation [4] required rapid-injection NMR techniques at very low temperatures. Without such techniques, however, a reactivity study may furnish dubious kinetic evidence if two or more equilibrium components in unknown proportions contribute to a global reaction rate. Therefore, it may be preferable to investigate (at least in opening studies) a purely monomeric species, in particular because this may be the most reactive form [2] that can dominate the reactivity profile of its mixture with aggregates. On the other hand, the analytic characterization of such a reactive species may be problematic due to a diminished kinetic stability against certain solvents, especially tetrahydrofuran (THF). For instance, barely avoidable decomposition products may interfere with traditional methods of molecular mass determinations that measure the averaged colligative properties of a solution. Fortunately, the ^13^C NMR techniques [5] exemplified further below can identify the ground-state structures of organolithiums even in partially decayed or contaminated solutions. However, the important problem of microsolvation in the coordination shell of lithium can normally not be addressed by NMR spectroscopy, because scrambling of coordinated and free ligands is usually so fast that only averaged resonances can be recorded even at low temperatures. Among the rare opposite cases are the very strong donor ligand (Me_2_N)_3_PO (HMPA) [6], or THF at the endocyclic Li of dimeric Me_2_CuLi&LiCN [7], or intramolecular (chelating) donor functions [8], whereas nonchelating monodentate ethereal donor ligands such as THF, Et_2_O, and *tert*-butyl methyl ether (*t-*BuOMe) normally show only the averaged signals. However, our sterically congested model system [9] **1** exhibited separate resonances of the latter three donor ligands (“Don” in Scheme 1) above the melting points of the solvent mixtures, so that the microsolvation numbers *d* could be measured by simple NMR integrations. On this basis, dimerization equilibria of **1a** and **1b** were analyzed [10] with a proper allowance for the differing microsolvation of the monomeric and dimeric components.

The alkenyllithiums **1a–e** and **2** are exclusively trisolvated (*d* = 3) monomers [9,11] in THF as the solvent, whereas **1a** forms purely disolvated (*d* = 2) monomers in Et_2_O, *t-*BuOMe, and 1,2-bis(dimethylamino)ethane (TMEDA). All dimeric species of **1a** and **1b** are disolvated (*d* = 1 at each Li) by the above three donor ligands in toluene solution and in the solid state [9]. These *d* values were found [9] to change inversely with the magnitude of the scalar one-bond ^13^C,^6^Li NMR coupling constants ^1^*J*_C,Li_ as quantified by the empirical Equation 1, where *n* is the number of Li cations coordinated to the carbanionic center ^13^C-α under consideration, while *a* is the number of C-α centers in direct contact with a certain Li cation; obviously, *n* = *a* = 1 for monomeric species such as **1** or **2**. Synthesized with the experimentally most convenient (because least line-broadening [12]) isotope ^6^Li, almost [9] all species of **1a** and **2** were found to have a common sensitivity factor of *L* = 42.8 Hz in Equation 1, whereas *L* decreased slightly in a nonlinear fashion with increasing Hammett parameters σ_p_^–^ for R in **1a–e** [13]. Numerically different *L* values apply to alkyl-, phenyl-, and alkynyllithiums [9].

[1]
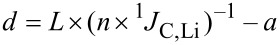


The ^13^C,^6^Li coupling constants ^1^*J*_C,Li_ can usually be detected only at sufficiently low temperatures where inter- and intramolecular scrambling of the ^6^Li cations becomes slow on the NMR time scales. Due to its spin quantum number *I* = 1, the single ^6^Li nuclear spin in a monomer (*n* = 1) will split the ^13^C-α resonance into 2*In* + 1 = 3 equally intense components. This 1:1:1 splitting pattern establishes a single ^13^C,Li contact, which provides evidence for the monomeric constitution of an organolithium compound that does not carry heteroatoms X as possible donor ligands for an intermolecular coordination (such as C–Li–X). The frequency intervals of this 1:1:1 triplet are numerically equal to the magnitude of ^1^*J*_C,Li_ which can reveal the degree of microsolvation via Equation 1: Decreasing (but nonzero) magnitudes of ^1^*J*_C,Li_ = *L* × [*n* (*a* + *d*)]^–1^ can result from increasing values of *d* (higher microsolvation). Using these two analytic tools of ^13^C-α splitting patterns and ^1^*J*_C,Li_ magnitudes, we will now establish the monomeric nature and the microsolvation numbers of the title compound which carries the small H_2_C= group in place of the bulky tetramethylindan-2-ylidene part of **1** and **2**. The latter constitutional difference was planned to become a touchstone for the cis/trans stereoinversion mechanism that was deduced earlier [1,11] for **1** and **2**.

## Results and Discussion

### Preparation and ground-state properties of **4**

**Scheme 2:**
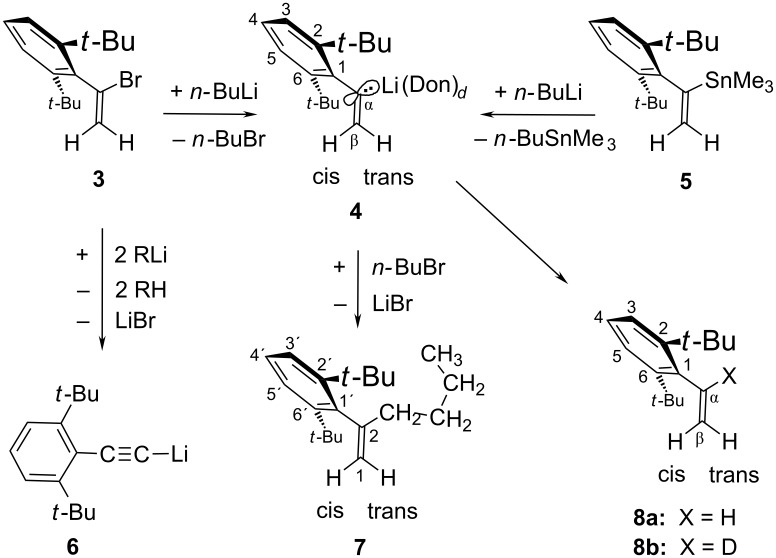
Preparation and derivatives of the α-arylvinyllithium **4**, where Don = ½TMEDA and *d* = 2 for the purified samples of **4**.

The Br/Li interchange reaction (Scheme 2) between *n-*BuLi and the bromoalkene [14] **3** that generates the title compound α-(2,6-di-*tert*-butylphenyl)vinyllithium (**4**) in cyclopentane as the solvent was almost finished after 70 min at room temperature (rt), affording 1-bromobutane (*n-*BuBr), the olefin [14] **8a**, the known [14] lithium arylacetylide **6**, and residual *n-*BuLi (by ^1^H NMR in situ analysis). Obviously, **4** had not reacted with its coproduct *n-*BuBr to produce **7**; instead, it had reacted faster than the concomitant *n-*BuLi to eliminate HBr from **3** and/or subsequently to deprotonate the generated arylalkyne [14] with formation of **6** and **8a**. After carboxylation and aqueous work-up, the acidic product fraction contained only the arylpropiolic acid (δ_H_ = 1.56 and 7.33 ppm in CDCl_3_) derived from **6** but not the α-arylacrylic acid to be expected from **4**.

In *t-*BuOMe as the solvent, bromoalkene **3** was consumed by *n-*BuLi in less than 20 min at rt, yielding roughly equivalent amounts of **4**, **6**, **8a**, and residual *n-*BuLi (the expected *n-*BuBr signal was perhaps overlaid). While *n-*BuLi vanished within 75 hours from this solution, the much slower decay of **4** at rt extended over more than 145 hours. Et_2_O as the solvent accelerated the Br/Li interchange reaction so much that a mixing problem emerged: While bromoalkene **3** was consumed by *n-*BuLi in less than five minutes at −70 °C and furnished mainly **4** along with a little of arylacetylide **6**, the slow introduction at or above −20 °C and faster local consumption of *n-*BuLi by a portion of **3** gave rise to a local accumulation of **4** in the presence of residual **3**, so that the elimination of HBr from **3** by **4** could produce comparable amounts of arylacetylide **6** and olefin **8a**. The finally surviving portion of **4** reacted very slowly (during >23 hours) at rt with its coproduct *n-*BuBr to yield hydrocarbon **7**. In THF as the solvent, such a mixing problem was encountered already at −70 °C: During the cautious addition of *n-*BuLi to bromoalkene **3**, **4** was generated and rapidly converted into **6**, **7**, and **8a** despite the presence of residual *n-*BuLi. In consideration of these various aspects of the reactivity profile of **4**, it became clear that sufficiently stable solutions of **4** could not be prepared from **3** directly.

**Figure 1:**
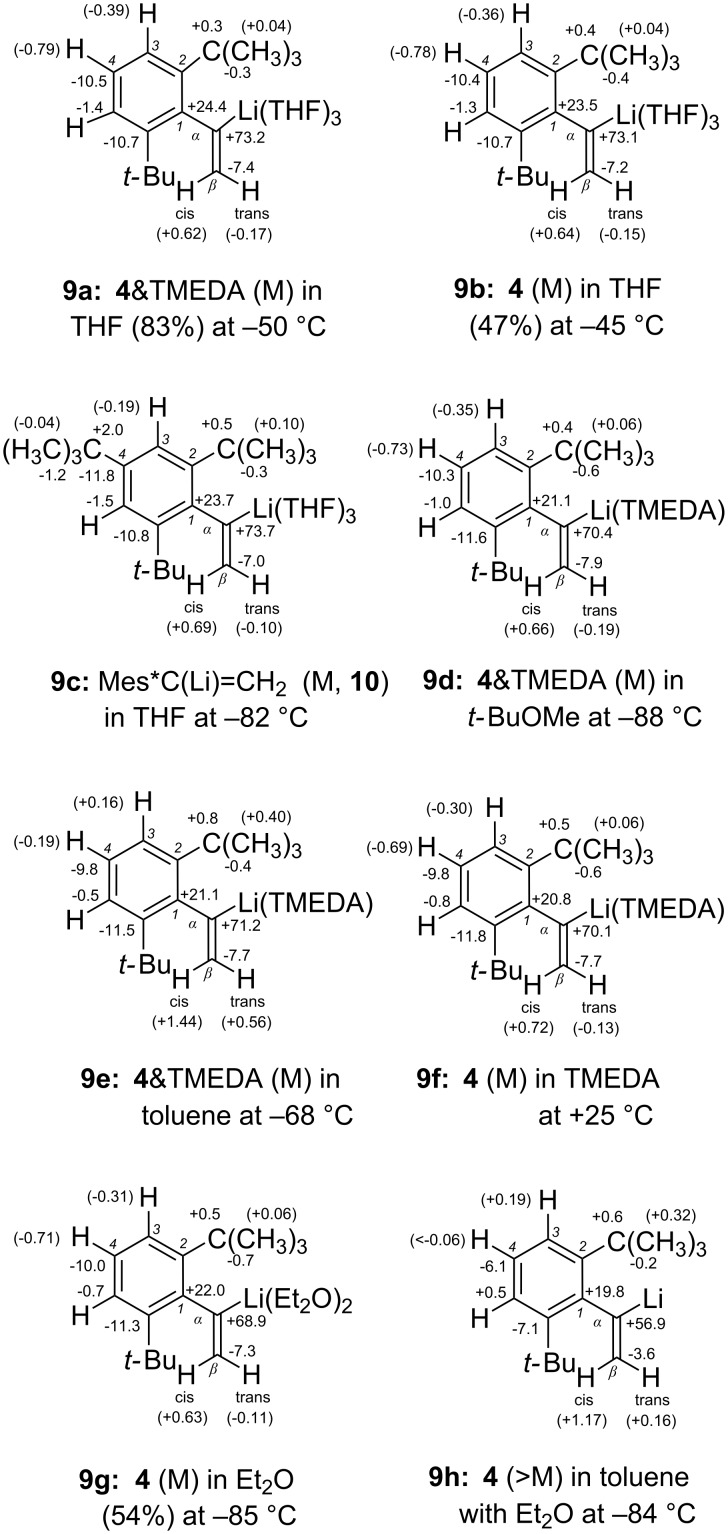
^13^C and (in parentheses) ^1^H NMR lithiation shifts Δδ = δ(R–Li) − δ(R–H) [ppm] of the monomers (“M”) **9a**–**g** of α-arylvinyllithiums **4** and **10** and the aggregate (“>M”) **9h** of **4**.

The successful detour preparation of more stable solutions of **4** made use of the trimethylstannyl precursor **5** that had been obtained [14] from bromoalkene **3** with LiSnMe_3_. A cyclopentane solution of **5** and *n-*BuLi did not react during several days at rt; on addition of TMEDA (ca. 3–5 equiv), however, merely one quickly recorded ^1^H NMR spectrum could be taken before crystals of **4**&TMEDA began to precipitate. Surplus *n-*BuLi converted the coproduct *n-*BuSnMe_3_ into *n-*Bu_2_SnMe_2_ (and sometimes into *n-*Bu_3_SnMe) along with a freely floating powder of MeLi. All such contaminations could be removed from the bunchy needles of **4**&TMEDA through washings with (cyclo)pentane, whereupon these purified crystals could be dissolved in an anhydrous solvent and stored under argon gas cover at −70 °C until NMR spectra could be measured. In such a colorless solution of **4**&TMEDA in THF/cyclopentane (ca. 83:17 by volume), THF had displaced TMEDA from its coordination to **4**; at below −50 °C, the olefinic proton resonances of **4** (Table S2, [15]) displayed a well resolved AB spectral system (two doublets) with the two-bond coupling constant ^2^*J*_H,H_ = 8.5 Hz (entry 2, Table 1). Conclusive evidence of the monomeric nature of [^6^Li]**4** arose from the ^13^C-α NMR triplet splitting (1:1:1) that became resolved also at and below −50 °C (Table S10, [15]). The magnitude of ^1^*J*_C,Li_ = 10.8 Hz indicated solvation by *d* = 3 THF ligands at Li, assuming that *L* = 42 (± 1) Hz in Equation 1 applies also to **4** (as will be confirmed further below). Practically equal ^1^H (Table S3, [15]) and ^13^C (Table S11, [15]) NMR data with identical magnitudes ^1^*J*_C,Li_ and ^2^*J*_H,H_ (entry 3, Table 1) were found for unpurified **4**&3THF that arose immediately from **5** with *n-*Bu^6^Li in the absence of TMEDA in a less clean (pale violet) THF/hydrocarbons mixture (47:53% by volume)_._ These coincidences established that even considerable amounts of residual *n-*Bu^6^Li and the emerging tetrameric Me^6^Li did not form mixed aggregates with **4** in THF. The further numerical similarity with ^1^*J*_C,Li_ = 10.7 Hz of **1a**&3THF [9] and **2**&3THF [9] (entry 1, Table 1; Don = THF and *d* = 3 in Scheme 1) is understood to indicate practically equal electronic properties (equal *L* parameters in Equation 1) of the C-α centers in all of these trisolvated monomers. As a topological requirement for that, the differing α-aryl groups in monomeric **1a** and **4** maintain almost orthogonal orientations with respect to the olefinic C=C double bond (as was also reported [14] for **3** and **5**). This orientation provides for an almost optimum delocalization of the σ-type (quasi-sp^2^) carbanionic electric charge at C-α of **4** (Scheme 2) into the α-aryl π-orbital system. The resulting negative lithiation NMR shifts Δδ = δ(**4**) − δ(**8a**) for ^13^C-4 and 4-H (entries 2 and 3, Table 1) point to a significant charge transfer [9,11] from C-α to C-4 of **4**&3THF. The full set of Δδ values of **4** in the two THF solutions is shown in formulae **9a** and **9b** of Figure 1, which provide a crude impression of the quasi-benzyl anion character of the α-aryl substituent with negatively charged *ortho* and *para* positions [16], although it must be admitted that signs and magnitudes of Δδ are not necessarily dominated by local electric charges in positions that are nearer than C-4 to the C–Li bond. Almost all lithiation shifts Δδ shown in **9c** (Figure 1) for α-(2,4,6-tri-*tert*-butylphenyl)vinyllithium (**10**) in THF [15] are closely similar to those of **4** in **9a** and **9b**, even though the chemical shifts δ(C-4) of **4** and **10** (Tables S11 and S12, [15]) differ by 20 ppm. Accordingly, **10** was established to be also a trisolvated monomer (^13^C-α triplet splitting) with characteristic values (entry 4, Table 1) of ^1^*J*_C,Li_ = 10.9 Hz, ^2^*J*_H,H_ = 8.6 Hz, and Δδ(C-α) = +73.7 ppm.

**Table 1:** Microsolvation numbers *d* and NMR data of α-(2,6-di- (**4**) and α-(2,4,6-tri-*tert*-butylphenyl)vinyllithium (**10**) with various solvents and donor ligands (Don) in comparison with the monomeric alkenyllithium **2**.^a^

entry	cpd no.	solvent (%)^b^	Don^c^ (equiv)	*d*	agg^d^	^1^*J*_C,Li_ [Hz]	^2^*J*_H,H_ [Hz]	Δ^2^*J*_H,H_ [Hz]	Δδ(C-α)	Δδ(C-4)	Δδ(4-H)	°C^e^

1	**2**	THF^f^	Solvent	3	M	t, 10.7	–	–	+66.0	−12.4	−0.88	−95
2	**4**	THF (83)^g^	Solvent	3	M	t, 10.8	8.5	6.3	+73.2	−10.5	−0.79	−50
3	**4**	THF (47)	Solvent	3	M	t, 10.8	8.5	6.3	+73.1	−10.4	−0.78	−45
4	**10**	THF	Solvent	3	M	t, 10.9	8.6	6.4	+73.7	−11.8	–	−82
5	**4**	*t*-BuOMe (77)	TMEDA (1.3)^h^	2	M	t, 13.9	7.4	5.1	+70.4	−10.3	−0.73	−88
6	**4**	toluene (85)	TMEDA (1.4)^i^	2	M	t, 13.8	7.4	5.2	+71.2	−9.8	−0.19^j^	−68
7	**4**	TMEDA (64)	solvent	2	(M)	–	7.4	5.1	+70.1	−9.8	−0.69	+25
8	**4**	Et_2_O (54)	solvent	2	M	t, 13.7	7.4	5.0	+68.9	−10.0	−0.71	−85
9	**4**	toluene (90)	Et_2_O (ca. 2)	?	>M	–	ca. 3.7	ca. 1.5	+56.9	−6.1	≈ −0.2^j^	−84

^a^t = triplet. ^b^Solvent % by volume, diluted with hydrocarbons. ^c^Possible donor ligands (equiv per Li). ^d^“M” = monomer, “>M” = unknown aggregate. ^e^Temperature of determination of Δδ = δ(R–Li) − δ(R–H) [ppm]. ^f^Table 1 of [11]. ^g^TMEDA (1–2 equiv) present but not coordinated. ^h^Microsolvation by TMEDA (1 equiv) detected at ≤ −68 °C. ^i^Microsolvation by TMEDA (1 equiv) detected at ≤ –44 °C. ^j^Temperature-dependent.

Both the clean (with TMEDA) and the contaminated (no TMEDA) THF solutions of **4**&3THF decayed at or above +3 °C through proton transfer from the solvent that was cleaved into ethylene and the enolate LiO–CH=CH_2_ [the latter identified through δ_H_ = 6.93 ppm (dd) and confirmed by δ_C_ = 81.9 and 158.9 ppm]. In *t*-BuOMe as the solvent, a purified sample of [^6^Li]**4**&TMEDA (entry 5, Table 1) displayed the triplet (1:1:1) of the ^13^C-α resonance already on cooling to −24 °C. The monomeric species identified by this triplet remained the only component of **4** between +25 and −88 °C, as documented by the practically temperature-independent ^1^H and ^13^C NMR chemical shifts (Tables S5 and S13, [15]). The increased magnitude of ^1^*J*_C,Li_ = 13.9 Hz (compared to 10.8 Hz in entries 2 and 3, Table 1) cannot be caused by the changed bulk solvent´s (*t-*BuOMe) macroscopic properties [9]; instead, it agrees with disolvation (*d* = 2 only) of a monomer for which *L* = 42 (± 1) Hz in Equation 1 predicts ^1^*J*_C,Li_ = 14 (± 0.3) Hz. This provided the above postponed confirmation that Equation 1 is valid in the **4** family. The NMR signals of TMEDA shifted and broadened on cooling and became split below −67 °C into separate absorptions of free and coordinated TMEDA (ca. 4:1). NMR integrations at −88 °C revealed the coordination of one molar equivalent of TMEDA. Although the severely broadened NCH_2_ and NCH_3_ signals did not enable us to differentiate between a symmetric (chelating) and an unsymmetrical (nonchelating) ligand binding of TMEDA at lithium, we are sure to have met the chelating mode which must be favored over the immoblization of additional ligands by its less negative entropy contribution. If so, this disolvation of monomeric **4** implies that TMEDA did not admit the solvent *t-*BuOMe to participate in direct microsolvation. After final quenching with DOCH_3_ (6 equiv), the in situ ^1^H and ^13^C NMR spectra showed the deuteriated olefin **8b** and displayed the expected signal shifting of TMEDA to the resonances of the free ligand.

The quasi-benzyl anion character of α-aryl in monomeric **4** is similar in the solvents THF (**9a** and **9b** in Figure 1), *t-*BuOMe with TMEDA (**9d**), toluene with TMEDA (**9e**), TMEDA (**9f**), and Et_2_O (**9g**). As above for **4**&TMEDA in *t-*BuOMe (entry 5, Table 1), the disolvated forms with the ligands TMEDA (in toluene) and Et_2_O (entries 6 and 8, Table 1) were established through the 1:1:1 triplet splittings of ^13^C-α and the magnitudes of ^1^*J*_C,Li_ = 13.8(1) Hz which are almost equal for the solvents *t-*BuOMe, toluene, and Et_2_O. As another striking observation, the magnitude of the olefinic ^2^*J*_H,H_ = 7.4 Hz for disolvation (entries 5–8, Table 1) is also independent of the kind of ligands and solvents. But why is it lower than for trisolvation by THF (entries 2–4, Table 1)? ^2^*J*_H,H_ coupling constants of olefinic =CH_2_ groups have been attributed [17–18] to σ-inductive substituent effects: More positive ^2^*J*_H,H_ values should be caused by increasing σ-electron donation through the molecular σ-orbital framework within the double-bond plane [17]. In this spirit, the ^2^*J*_H,H_ values of our model system **4** indicate that the sp^2^-type electron pair at C-α (Scheme 1 or Scheme 2) is a somewhat stronger σ-electron donor if coordinated to Li^+^(THF)_3_ than if coordinated to Li^+^(Et_2_O)_2_ or to Li^+^(TMEDA). Of course, the accompanying σ-inductive effect of the α-aryl group contributes likewise to ^2^*J*_H,H_ in both **4** and the “parent” olefin **8a**. Therefore, this σ-inductive “contamination” may be removed by subtraction, as described previously [18] for many other α-substituents: The differences Δ^2^*J*_H,H_ = ^2^*J*_H,H_(**4**) − ^2^*J*_H,H_(**8a**) were measured in situ and are included in Table 1 so as to present “purified” lithiation-induced contributions to the ^2^*J*_H,H_ values of **4**.

The diverse experimental setups for entries 6–8 provided the following additional information. Purified [^6^Li]**4**&TMEDA in [D_8_]toluene/cyclopentane (85:15 by volume, entry 6) occurred as a single species between +25 and −82 °C, as shown by the temperature-independent ^13^C NMR data (Table S14, [15]), whereas the notoriously variable [19] ^1^H NMR data evaded a simple interpretation. Importantly, separate NMR signals were observed for free and coordinated TMEDA (1 equiv) at and below −44 °C. In TMEDA/hydrocarbons (64:36) as the solvent, the quick Sn/Li interchange reaction of **5** with *n-*Bu^6^Li at rt furnished both [^6^Li]**4** and Me^6^Li as explained further above. The assignment as a disolvated monomer ["(M)" in entry 7, Table 1] had to rely on ^2^*J*_H,H_ and Δδ values (entry 7, Table 1 and **9f**), because **4**&TMEDA began to precipitate at −22 °C, which made ^1^*J*_C,Li_ evidence unavailable (Tables S7 and S15, [15]). The high concentration of (CH_3_Li)_4_ did not change during the slow decay of **4**&TMEDA at rt through proton transfer from the solvent TMEDA [20–21] that formed olefin **8a**, Me_2_N–CH=CH_2_, and LiNMe_2_. In Et_2_O/hydrocarbons (54:46) without TMEDA, the Sn/Li interchange reaction of **5** and *n-*Bu^6^Li was very slow with a first half-life time of ca. 21 hours at rt. As the hitherto only case among these species of **4**, we met here a weak aberration from the property of temperature-independent δ values: Most ^13^C and some ^1^H NMR chemical shifts, which characterize the disolvated monomer **4**&2Et_2_O (entry 8, Table 1), began to change slightly (Tables S8 and S16, [15]) during warm-up in the direction expected [22] for aggregation. The suspicion that *n-*BuLi might be involved was supported through comparison with a purified sample of **4**&TMEDA in Et_2_O/[D_12_]cyclohexane (80:14) that contained neither *n-*BuLi nor MeLi: Now the ^1^H (at +11 and +25 °C in Table S8, [15]) and ^13^C δ values (at +11 and −10 °C in Table S16, [15]) were no longer different from those of monomeric **4**&2Et_2_O, and the concomitant TMEDA exhibited the NMR signals of the free ligand.

Returning to the above aberration of unpurified **4** in Et_2_O (without TMEDA, but contaminated by *n-*BuLi and MeLi), we searched for more convincing evidence of aggregating **4** as follows. After evaporation of the solvent under a reduced pressure of dry argon gas, the remaining oil (ca. 0.02 mL) dissolved readily in [D_8_]toluene, except for an insoluble powder of (CH_3_Li)_4_. This solution contained [^6^Li]**4** along with Et_2_O (ca. 2 equiv), *n-*Bu^6^Li, olefin **8a**, and a portion of the starting material **5** with partially modified α-trialkylstannyl (*n-*Bu_x_Me_3–x_Sn) groups. Although a ^1^*J*_C,Li_ coupling could not be resolved in this experiment (Table S17, [15]), the significantly diminished quasi-benzyl anion character shown in **9h** (Figure 1) indicated that this form of [^6^Li]**4** was no longer the monomer but must have become aggregated. In particular, the magnitudes of Δδ(C-α) and Δδ(C-4) in entry 9 of Table 1 were smaller than those in entry 8 by decrements of 12 and 4 ppm, respectively, in agreement with the corresponding effects of dimerization that were reported [22] for **1a** in toluene. A ^1^H NMR spectrum taken at −55 °C revealed a substantially decreased magnitude of ^2^*J*_H,H_ = ca. 3.7 Hz. The lack of further ^2^*J*_H,H_ data in Table S9 [15] was due to strong line broadening in the presence of residual *n-*Bu^6^Li. Apart from excluding the monomer, these results suggested that may be able to form mixed aggregates if Et_2_O ligands are in short supply.

### The pseudomonomolecular, ionic cis/trans sp^2^-stereoinversion of α-(2,6-di-*tert*-butylphenyl)vinyllithium (**4**)

**Scheme 3:**
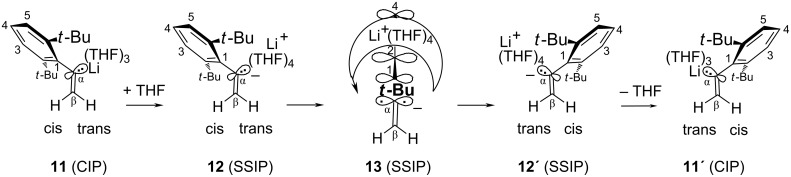
THF-catalyzed ionization of ground-state **11** (CIP) generates the solvent-separated ion pair **12** (SSIP), whose sp^2^-stereoinversion via a more polar transition state **13** (SSIP) [11] occurs with migration of Li^+^(THF)_4_ and is followed by the release of THF from **12'** (SSIP) to form the stereoinverted ground-state **11'** (CIP).

In a stereochemically “frozen” ground-state of **4**, the olefinic protons H-cis and H-trans give rise to two NMR doublets (“AB” spectral system) with the above-mentioned geminal coupling constant ^2^*J*_H,H_. These two “diastereotopic” protons (which differ stereochemically but are equivalent by connectivity) can interchange their environments (cis and trans) through a “diastereotopomerization” [23] that interconverts the ground-state contact-ion pairs (CIP) **11** and **11'** in Scheme 3. Increasing rates of this interchange will cause at first an increasing line broadening of the AB system and then a “coalescence” of all four lines into a singlet at the averaged resonance position of (δ_cis_ + δ_trans_)/2 [24]. The temperature-dependent pseudo-first-order rate constants *k*_ψ_ of this interconversion were determined with a computer program [25–28] that can simulate and plot AB line shapes for visual comparison with the experimental spectra. This technique demands extrapolating the values of δ_cis_ – δ_trans_ and ^2^*J*_H,H_ from a series of low-temperature (less broadened) AB spectra into the coalescence domain.

**Figure 2:**
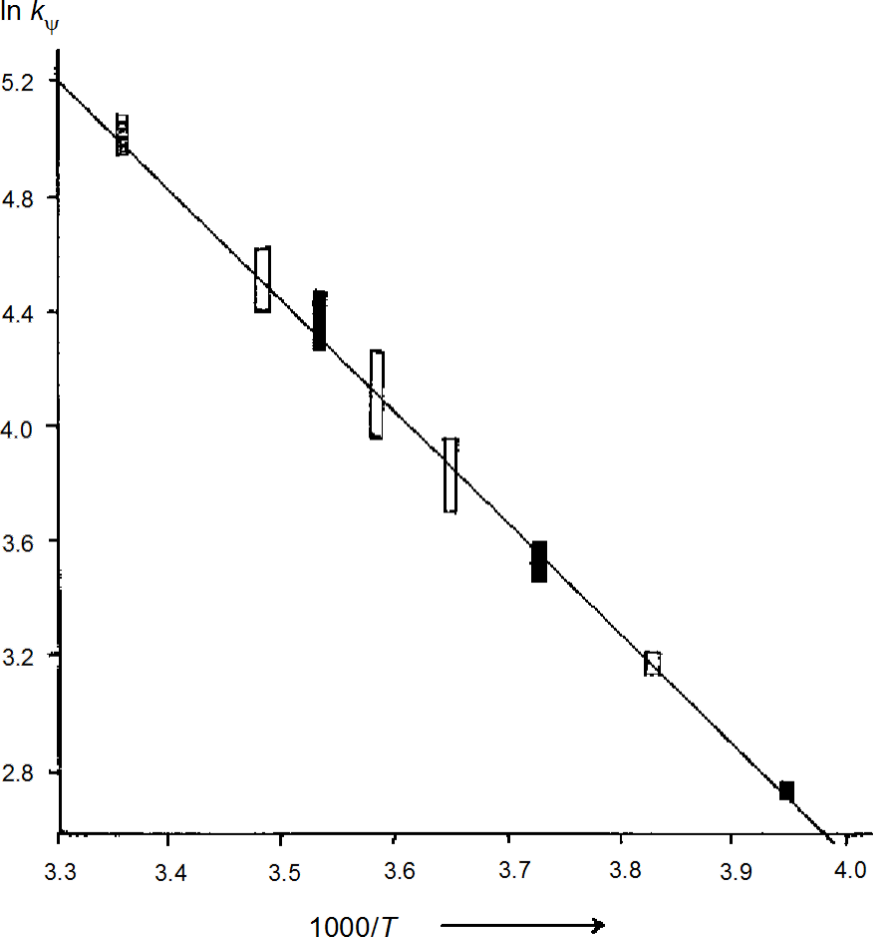
Arrhenius diagram of the natural logarithms of pseudo-first-order rate constants *k*_ψ_ [s^−1^] of sp^2^-stereoinversion versus 1000/*T* [K^−1^] for **4** in THF (47%) solution. Concentrations of **4**: open symbols, 0.09 M; hatched, 0.13 M; filled, 0.17 M.

The temperature dependence of the resultant rate constants *k*_ψ_ (Table S1, [15]) is quantified in Figure 2 by a single straight line, which established that *k*_ψ_ did not depend on the concentrations of **4** in THF (47% by volume). Therefore, the diastereotopomerization mechanism can be neither associative (for example, bimolecular) nor dissociative (generating the free ions from **4**); instead, the rates depend on the concentration of the substrate **4** in a first order as defined in Equation 2. This important kinetic property raises the problem of devising a monomolecular (yet nondissociative) manner of cleaving the C–Li bond in preparation of the stereoinversion step. An obvious possibility consists in the transient heterolysis with formation of an NMR-invisible, solvent-separated ion pair (SSIP) **12** in Scheme 3. Such a heterolysis must be induced by the transitory immobilization of a THF ligand, which will be released on formation of the inverted product **11'** via the diastereotopomer **12'** of **12**. Thus, the sp^2^-stereoinverion process is catalyzed by THF, so that its pseudo-first-order rate constant *k*_ψ_ in Equation 2 is the product of a second-order rate constant *k*_0_ and the constant concentration of free THF. One might be tempted to call on Equation 2 for interpreting the twofold increase of *k*_ψ_ that was observed for **4** in a more concentrated (85% in place of 47%) THF solution (−8 °C in Table S2, [15]); however, this weak acceleration might also be due to the increased solvent polarity.

[2]
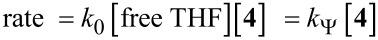


The pseudomonomolecular, ionic mechanism in Scheme 3 had previously [11] been deduced through finding a Hammett reaction constant of ρ = +5.2 for the sp^2^-stereoinversions of the paradigm family of **1a**–**e**, which established that charge separation increased substantially on the way to the transition state (corresponding to **13**). In a similar process, C-α in **13** should become nearly sp-hybridized (linear) and more distant from Li^+^ than it is in **11**. Consequently, the negative charge at C-α in **13** will be delocalized into the aromatic π-electron system more efficiently than in **11** and **12**, so that the quasi-benzyl anion character and the concomitant charge separation increase on the way from **11** to **13**. Li^+^(THF)_4_ can then utilize the increasing charge gradients to perform the required migration shown in **13** during stereoinversion of the carbanion, as had been postulated [11] for **1**. This proposal is now supported in Table 2 through the similar pseudoactivation parameters of **4**&3THF (entry 3, Table 2), **2**&3THF (entry 2, Table 2), and **1a**&3THF (entry 1, Table 2). Although migration across the β-unsubstituted H_2_C= region in **13** might be sterically much more comfortable than across the bulky β,β-di-*tert*-alkyl substituents in the transition states deriving from **1** or **2**, this opportunity is apparently disdained in **13**: The stereoinversion is not facilitated for **4**, since the Δ*G*_ψ_^‡^ barrier of **4** is slightly higher than those of **1a** and **2**, which may be caused by a structure-dependent decrease of the ground-state free enthalpies (*G*) that is somewhat stronger for **4**. We conclude that the migration of Li^+^(THF)_4_ in **13** occurs likewise away from the C-β region, namely, toward the negative charge center C-4 of the orthogonally oriented α-aryl group without being seriously impeded by the two *t*-Bu groups. With a 4-*t-*Bu substituent in **10** [15], however, the corresponding diastereotopomerization was found to be roughly threefold retarded (albeit not quantified because of decomposition); this may have been caused by the decelerating (because electron-donating) effect of 4-*t*-Bu (σ_p_^–^ = −0.13 [29–30]) in combination with some steric hindrance of the Li^+^(THF)_4_ migration past 4-*t*-Bu, even though a concomitant accelerating effect might be expected from a somewhat stronger negative charge at C-4 in **10** (compare the ground-state **9c** with **9a** in Figure 1). Altogether, these considerations argue for the possibility that Li^+^(THF)_4_ circumnavigates 3-H (or 5-H) in **13**, conducted by the negative charges in α-aryl.

**Table 2:** Pseudoactivation parameters Δ*G*_ψ_^‡^ (kcal mol^−1^ at 0 °C), Δ*H*_ψ_^‡^ (kcal mol^−1^), and Δ*S*_ψ_^‡^ (cal mol^−1^ K^−1^) of cis/trans diastereotopomerization rates of three monomeric 1-aryl-1-alkenyllithiums (**1a**, **2**, and **4**) in THF.

entry	cpd no.	aryl substituent	Δ*G*_ψ_^‡^ (0 °C)	Δ*H*_ψ_^‡^	Δ*S*_ψ_^‡^	reference

1	**1a**	4´-H	13.35 ± 0.03	6.63 ± 0.24	−24.6 ± 1.0	[11]
2	**2**	2’,6’-CH_3_	12.47 ± 0.01	6.77 ± 0.18	−20.8 ± 0.7	[11]
3	**4**	2’,6’-*t*-Bu	13.867 ± 0.002	7.02 ± 0.09	−25.1 ± 0.4	this work

As outlined previously [11], the trisolvation privilege of THF facilitates the sp^2^-stereoinversion through limiting the negative entropy contribution of ligand immoblization: Since **4**&3THF needs only one further THF ligand to generate the SSIPs **12** and **13** from **11**, this immobilization costs only a one-particle entropy contribution of ca. −11 cal mol^−1^ K^−1^ [10], as also known [11] for the diastereotopomerization of **1a**&3THF and **2**&3THF whose Δ*S*_ψ_^‡^ values (entries 1 and 2, Table 2) are similar to those of **4**&3THF (entry 3, Table 2), which suggests a similar course of the stereoinversion processes. In contrast, the disolvated monomers **4**&2*t*-BuOMe and **4**&2Et_2_O would have to immobilize two additional ligands at the expense of doubled entropy contributions (ca. −22 cal mol^−1^ K^−1^) if generating SSIPs (like **12**) with tetrasolvated Li^+^, which explains why their diastereotopomerization rates remained below our NMR time scales even though the doubled THF fixation would provide an increased negative contribution to Δ*H*_ψ_^‡^. For a critical evaluation of these pseudoactivation parameters, it may be recalled [10–11] that the “true” activation enthalpies Δ*H*^‡^ will be almost equal to Δ*H*_ψ_^‡^, whereas true Δ*S*^‡^ will be more negative than Δ*S*_ψ_^‡^ by a mathematical correction of roughly *R* ln[free THF] = up to 5 cal mol^−1^ K^−1^.

## Conclusion

α-(2,6-Di-*tert*-butylphenyl)vinyllithium (**4**) and its 4-*t*-Bu congener (**10**) are the first β-unsubstituted vinyllithiums whose microsolvation numbers in solution could be established directly by NMR integrations and/or through ^1^*J*_C,Li_ values via the empirical Equation 1. This became possible through two-sided shielding of the C–Li part of **4** by two *tert*-butyl substituents: these gave rise to unusually low rates of donor ligand exchange at lithium and of some reactions with **4**, and they caused also a very weak inclination of monomeric **4** to aggregate in solution, in contrast to the majority of known H_2_C=C(Li)–R species. Such conditions enabled us to establish also the magnitude of the olefinic ^2^*J*_H,H_ coupling constant as a secondary criterion for the degree of microsolvation: Independent of the kind of ligands and solvents (Table 1), the values were 8.5 Hz for trisolvated and 7.4 Hz for disolvated monomers.

The rapid cis/trans stereoinversion of monomeric **4** in THF provides a possible explanation of the stereorandom formation [14] of [β-D]**5** through Br/Sn interchange and is partially due to trisolvation of the ground-state: The necessary C–Li bond heterolysis is achieved through immobilization of only one further THF ligand at lithium with a correspondingly low entropic penalty (ca. 44% of Δ*S*_ψ_^‡^). The energetic expenditure also remains low (ca. 50% of Δ*G*_ψ_^‡^ at 0 °C) because the carbanion and Li^+^(THF)_4_ do not dissociate from the solvent-separated ion pair and because the α-aryl substituent stabilizes the carbanionic charge through an increasingly efficient quasi-benzyl anion resonance in the transition state **13**. The close agreement of these pseudoactivation parameters of **4** with those of **2** and **1a** suggests that the migration of Li^+^(THF)_4_ within the solvent-separated ion pair does not take place across the H_2_C= region of **4** since this region is obstructed in **1** and **2** which nevertheless show closely similar pseudoactivation parameters as **4**.

## Experimental

**General remarks.** An NMR tube (5 mm) containing the α-arylvinyllithium **4** or **10** together with a donor ligand in [D_8_]toluene or in a nondeuteriated solvent [with [D_12_]cyclohexane (0.060 mL) as the lock substance] with a trace of TMS under argon gas cover was sealed with a soft, solvent-resistant rubber stopper that was finally wrapped with parafilm^®^. Customary methanol NMR tubes were measured in place of the sample tubes to determine the actual spectrometer temperatures. ^1^H and ^13^C NMR chemical shifts δ [ppm] were referenced to internal TMS. The experimental rate constants *k*_ψ_ and their error limits were obtained from the line shapes of strongly expanded ^1^H NMR spectral regions through visual comparison with those of computed [25] spectra. Natural logarithms (ln) refer to the dimensionless magnitudes of the employed quantities.

**[^6^Li]-α-(2,6-Di-*tert*-butylphenyl)vinyllithium ([^6^Li]4).** A dried NMR tube (5 mm) was charged with the trimethylstannyl alkene [14] **5** (51 mg, 0.13 mmol), dry cyclopentane (0.60 mL), and TMEDA (0.068 mL, 0.45 mmol), then cooled under argon gas cover in an ice-bath during the addition of *n-*Bu^6^Li (0.15 mmol) in cyclopentane (0.060 mL). The tightly closed tube was stored in a large Schlenk tube filled with argon gas at −30 °C until the following washing procedure: The supernatant was withdrawn from the crystals of **4**&TMEDA by syringe under argon gas; fresh dry cyclopentane (0.3 mL) was added, the tube was gently shaken, and the crystals were allowed to settle in the tube. After one or more repetitions of such washings and final withdrawal, the crystals were blown dry in a soft stream of dried argon gas for ca. five seconds, then dissolved in the appropriate anhydrous solvent (0.5 mL) together with TMS for NMR measurements [15]. ^1^H NMR of **4**&TMEDA (Et_2_O, 400 MHz, expansion of the shortened presentation at 25 and 11 °C in Table S8 [15]) δ 1.44 (s, ca. 18H, 2-/6-CMe_3_), 5.31 (d, ^2^*J* = 7.4 Hz, 1H, β-H trans to aryl), 5.64 (d, ^2^*J* = 7.4 Hz, 1H, β-H cis), 6.34 (t, ^3^*J* = 7.8 Hz, 1H, 4-H), 6.95 (d, ^3^*J* = 7.8 Hz, 2H, 3-/5-H) ppm; ^13^C NMR of **4**&TMEDA (Et_2_O, 100.6 MHz, at −10 °C with CH coupling constants for Table S16 [15]) δ 32.1 (qm, ^1^*J* = 125 Hz, ^3^*J* = 4.9 Hz, 2-/6-C*Me*_3_), 37.7 (unresolved, 2-/6-C), 112.0 (t, ^1^*J* = ca. 148 Hz, C-β), 117.0 (sharp d, ^1^*J* = 156 Hz, C-4), 123.9 (dd, ^1^*J* = 150 Hz, C-3/-5), 137.4 (unresolved, C-2/-6) ppm, C-α and C-*ipso* not detected because of low solubility. ^1^H NMR of **4**&TMEDA (cyclopentane, 200 MHz, 23 °C) δ 1.44 (s, Δδ = +0.06 ppm, 2-/6-CMe_3_), 5.28 (d, ^2^*J* = 7.0 Hz, Δ^2^*J* = 4.6 Hz, Δδ = −0.09 ppm, 1H, β-H trans to aryl), 5.67 (d, ^2^*J* = 7.0 Hz, Δδ = +0.74 ppm, 1H, β-H cis to aryl), 6.35 (t, ^3^*J* = 7.8 Hz, Δδ = −0.60 ppm, 1H, 4-H), 6.95 (t, ^3^*J* = 7.8 Hz, Δδ = −0.28 ppm, 2H, 3-/5-H), TMEDA (7 equiv) at 2.21 and 2.13 ppm.

**2-(2´,6´-Di-*tert*-butylphenyl)hex-1-ene (7).** A solution of bromoalkene **3** (54 mg, 0.18 mmol) [14] in anhydrous THF (0.50 mL) was placed in an NMR tube (5 mm) and cooled at −70 °C under argon gas cover during the addition of *n-*BuLi (0.20 mmol) in hexane (0.10 mL). After 120 min at −70 °C, the dark red solution was poured onto solid CO_2_, warmed up, and diluted with Et_2_O (10 mL) and aqueous NaOH (20 mmol, 10 mL). The aqueous layer was extracted with Et_2_O (10 mL); both Et_2_O extracts were combined, washed with dist. water until neutral, dried over MgSO_4_, and concentrated. The remaining crude oil (24 mg) was a mixture of **7** and the olefin **8a**. ^1^H NMR of **7** (CDCl_3_, 400 MHz) δ 0.94 (t, ^3^*J* = 7.3 Hz, 3H, 6-CH_3_), 1.40 (s, 18H, 2´-/6´-CMe_3_), ca. 1.4 (overlaid, 5-CH_2_), ca. 1.58 (m, ^3^*J* = ca. 8 Hz, 2H, 4-CH_2_), 2.24 (tdd, ^3^*J* = 8 Hz, 2H, 3-CH_2_), 5.20 (dt, ^2^*J* = 1.8 Hz, ^4^*J* = 1.6 Hz, 1H, 1-H cis to aryl), 5.36 (dt, ^2^*J* = 1.8 Hz, ^4^*J* = 1.9 Hz, 1H, 1-H trans), 7.12 (t, ^3^*J* = 8.0 Hz, 1H, 4´-H), 7.39 (d, ^3^*J* = 8.0 Hz, 2H, 3’-/5’-H) ppm; ^13^C NMR of **7** (CDCl_3_, 100.6 MHz) δ 14.1 (qqi, ^1^*J* = 124.2 Hz, │^2^*J* + ^3^*J*│/2 = 4 Hz to CH_2_CH_2_, 6-*C*H_3_), 22.6 (tm, ^1^*J* = ca. 123 Hz, 5-*C*H_2_), 28.2 (tm, ^1^*J* = 123 Hz, 4-*C*H_2_), 33.3 (qm, ^1^*J* = 125.4 Hz, ^3^*J* = 4.9 Hz, 2’-/6’-C*Me*_3_), 38.0 (m, quart. 2’-/6’-C), 41.0 (tm, ^1^*J* = 123.5 Hz, apparent *J* = 4.5 Hz to CH_2_CH_2_, 3-*C*H_2_), 115.9 (ddt, ^1^*J* = 157 and 159 Hz, ^3^*J* = 4.6 Hz, C-1), 125.9 (ddd, ^1^*J* = 155 Hz, ^3^*J* = 7.5 Hz, ^2^*J* = 2.3 Hz, C-3’/-5’), 126.0 (sharp d, ^1^*J* = 157.5 Hz, C-4’), 143.2 (unresolved, C-1’), 147.2 (dm, ^3^*J* = 7.1 Hz, C-2’/-6’), 150.4 (broadened t, ^2^*J* = 6.5 Hz, C-2) ppm, assigned through selective {^1^H} decoupling as follows: {C(C*H*_3_)_3_} → C-2’/-6’ as a sharp d with ^3^*J* = 7.1 Hz, quart. 2’-/6’-C as the X part of an ABX system with ^3^*J* + ^5^*J* = 4.2 Hz; {C*H*_2_-3} → C-1 as a dd, C-2 as a broadened s.

**2,6-Di-*tert*-butylstyrene (8a)** [14]. ^2^*J*_H,H_ = 2.2 Hz in CDCl_3_ and THF; 2.3 Hz in cyclopentane, TMEDA/toluene, and TMEDA/*t-*BuOMe; 2.4 Hz in Et_2_O and toluene.

**[α-D]-2-/6-Di-*tert*-butylstyrene (8b).** This was obtained through the addition of DOCH_3_ (0.5 mmol) to a solution of **4**&TMEDA in *t-*BuOMe that is documented in Tables S5 and S13 [15]. ^1^H NMR (*t-*BuOMe/cyclopentane, 400 MHz, 25 °C) δ 1.38 (s, 18H, 2-/6-CMe_3_), 4.93 (dt, ^2^*J* = 2.3 Hz, 1H, β-H cis to aryl), 5.39 (dt, ^2^*J* = 2.3 Hz, 1H, β-H trans), 7.04 (t, ^3^*J* = 8.0 Hz, 1H, 4-H), 7.25 (d, ^3^*J* = 8.0 Hz, 2H, 3-/5-H) ppm; ^1^H NMR (CDCl_3_, 400 MHz) δ 1.40 (s, │^6^Δ│ < 0.0015 ppm, 18H, 2-/6-CMe_3_), 5.005 (dt, ^2^*J* = 2.2 Hz, ^3^*J*_HD_ = 2.8 Hz, ^3^Δ = −0.0085 ppm, β-H cis to aryl), 5.449 (dt, ^2^*J* = 2.2 Hz, ^3^*J*_HD_ = 1.8 Hz, ^3^Δ = −0.0030 ppm, β-H trans), 7.15 (t, ^3^*J* = 8.0 Hz, 1H, 4-H), 7.34 (d, ^3^*J* = 8.0 Hz, 2H, 3-/5-H) ppm; ^13^C NMR (*t-*BuOMe/cyclopentane, 100.6 MHz, 25 °C) δ 32.86 (2-/6-C*Me*_3_), 37.28 (quart. 2-/6-C), 119.536 (^2^Δ = –0.133 ppm, C-β), 124.70 (C-3/-5), 127.05 (C-4), 140.0 (^2^Δ = −0.090 ppm, C-1), 141.47 (t, ^1^*J*_CD_ = 23.7 Hz, ^1^Δ = −0.318 ppm, C-α), 149.30 (C-2/-6) ppm; ^13^C NMR (CDCl_3_, 100.6 MHz) δ 32.30 [qm, ^5^Δ = −0.0067(3) ppm, 2-/6-C*Me*_3_], 36.79 [^4^Δ = −0.0056(3) ppm, quart. 2-/6-C], 119.22 [sharp t, ^1^*J* = 157 Hz, ^2^*J*_CD_ = 0 Hz [31], ^2^Δ = −0.134(4) ppm, C-β], 124.20 [dm, ^4^Δ = −0.0103(2) ppm, C-3/-5], 126.236 [d, ^5^Δ = +0.0065(7) ppm, C-4], 139.65 [m, ^2^Δ = −0.0845(8) ppm, C-1], 140.05 [t, ^1^*J*_CD_ = 24.0 Hz, ^1^Δ = −0.321(2) ppm, C-α], 148.995 [m, ^3^Δ = +0.0315(6) ppm, C-2/-6] ppm.

## Supporting Information

File 1 Preparation, properties, and derivatives of α-(2,4,6-tri-*tert*-butylphenyl)vinyllithium (**10**); Table S1 of diastereotopomerization rate constants; Tables S2–S17 of primary NMR data.
