# Supplementary material for: Microsolvation and sp2-stereoinversion of monomeric α-(2,6-di-tert-butylphenyl)vinyllithium as measured by NMR
Source: Beilstein J Org Chem. 2014 Oct 29;10:2521–30. doi: 10.3762/bjoc.10.263 (PMC4222283; doi:10.3762/bjoc.10.263)
Supplement: File 1 — Preparation, properties, and derivatives of α-(2,4,6-tri-tert-butylphenyl)vinyllithium (10); Table S1 of diastereotopomerization rate constants; Tables S2–S17 of primary NMR data. [file Beilstein_J_Org_Chem-10-2521-s001.pdf]

Supporting Information

for

Microsolvation and sp^2-stereoinversion of monomeric α-(2,6-di-*tert*-butylphenyl)vinyllithium as measured by NMR

Rudolf Knorr*, Monika Knittl and Eva C. Rossmann

Address: Department Chemie, Ludwig-Maximilians-Universität München, Butenandtstrasse 5–13 (Haus F), 81377 München, Germany

Email: Rudolf Knorr* - rhk@cup.uni-muenchen.de

* Corresponding author

Preparation, properties, and derivatives of α-(2,4,6-tri-*tert*-butylphenyl)vinyllithium (10); Table S1 of diastereotopomerization rate constants; Tables S2–S17 of primary NMR data.

Contents

1. **α-(2,4,6-Tri-*tert*-butylphenyl)vinyllithium (10)** page S3

   Preparation, properties, and derivatives **S5** and **S7** page S3

2. **References** page S5

3. ***Cis*/*trans* diastereotopomerization rate constants** (Table S1) page S6

4. **Tabulated primary NMR data**

   Table S2. Temperature dependence of δ_H for monomeric **4**&TMEDA in THF (83%) page S6

   Table S3. Temperature dependence of δ_H for monomeric **4** in THF (47%) page S7

   Table S4. Temperature dependence of δ_H for monomeric **10** in THF page S7

   Table S5. Temperature dependence of δ_H for monomeric **4**&TMEDA in *t*BuOMe page S8

   Table S6. Temperature dependence of δ_H for monomeric **4**&TMEDA in [D_8]toluene page S8

   Table S7. Temperature dependence of δ_H for monomeric **4** in TMEDA page S9

   Table S8. Temperature dependence of δ_H for monomeric **4** in Et_2O (54 and 80%) page S9

   Table S9. Temperature dependence of δ_H for aggregated **4**&Et_2O in [D_8]toluene page S10

   Table S10. Temperature dependence of δ_C for monomeric **4**&TMEDA in THF (83%) page S10

   Table S11. Temperature dependence of δ_C for monomeric **4** in THF (47%) page S11

   Table S12. Temperature dependence of δ_C for monomeric **10** in THF page S11

   Table S13. Temperature dependence of δ_C for monomeric **4**&TMEDA in *t*BuOMe page S11

   Table S14. Temperature dependence of δ_C for monomeric **4**&TMEDA in [D_8]toluene page S12

   Table S15. Temperature dependence of δ_C for monomeric **4** in TMEDA page S12

   Table S16. Temperature dependence of δ_C for monomeric **4** in Et_2O (54 and 80%) page S13

   Table S17. Temperature dependence of δ_C for aggregated **4**&Et_2O in [D_8]toluene page S13

1. α-(2,4,6-Tri-*tert*-butylphenyl)vinyllithium (10).

Preparation and properties. In spite of the two-sided shielding [S1] of the bromoalkene **S1**, its Br/Li interchange reaction with *n*-BuLi (Scheme S1) occurred rapidly in THF, Et_2O, or *t*-BuOMe at low temperatures with formation of **10** and 1-bromobutane (*n*-BuBr). These high rates created a mixing problem during the addition of *n*-BuLi to **S1** in NMR tubes: After the local consumption of *n*-BuLi by a portion of the locally present **S1**, up to two equivalents of the just generated **10** were consumed in eliminating HBr from residual **S1** and in the subsequent deprotonation of the emerging arylalkyne, forming the arylacetylide **S3** along with up to two equivalents of the known [S1] olefin **S6**. This was recognized through carboxylation with solid CO_2, which furnished the arylpropiolic acid [S1] **S4** (from **S3**) and the α-arylacrylic acid **S7** (from residual **10**). Before or without such a carboxylation, **10** reacted with its coproduct *n*-BuBr immediately in THF solution but slowly in Et_2O or in *t*-BuOMe. Therefore, this butylation of **10** was avoided in Et_2O (though not in THF) by the addition of *n*-BuLi (1.3 equiv) to **S1** at or below −50 °C and quick mixing, followed by rapid warm-up and careful evaporation of all volatile compounds (including *n*-BuBr) under a falling pressure of dry argon gas down to 0.01 mbar. The pot residue contained nonvolatile (because coordinated) Et_2O but did not crystallize; it was dissolved in THF, Et_2O, or *t*-BuOMe.

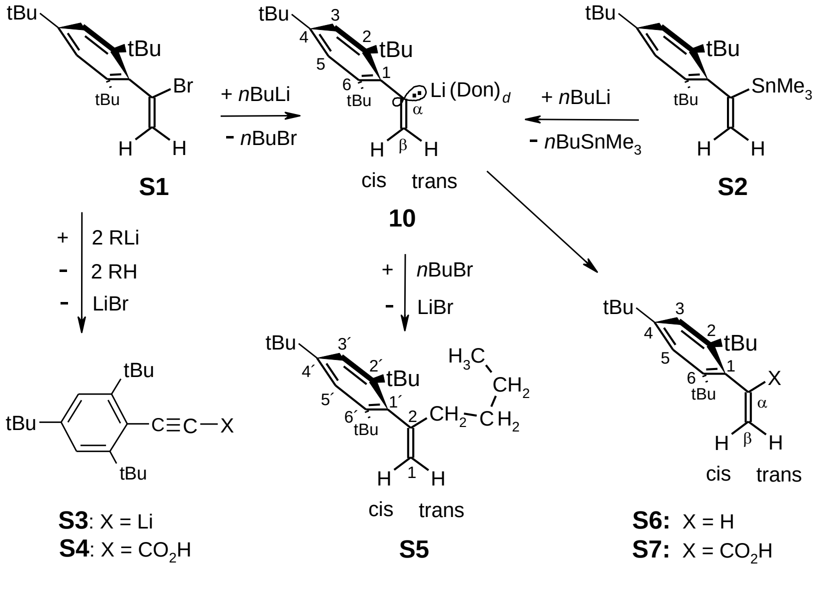

Scheme S1: Preparation and derivatives of α-(2,4,6-tri-*tert*-butylphenyl)vinyllithium (**10**).

10 is a trisolvated monomer in THF solution, as established by the triplet (1:1:1) splitting of its ^{13}C-α NMR resonance by one ^{6}Li nuclear spin; the magnitude 10.9 Hz of its scalar one-bond coupling constant $^{1}J_{CLi}$ at −82 °C revealed microsolvation by d = 3 THF ligands at lithium according to the empirical equation 1 in the Main Text. This monomer was the only species of **10** in THF between −82

and +3 °C since its ^{1}H and ^{13}C NMR chemical shifts δ (Tables S4 and S12) did not depend on the temperature. The cis/trans stereoinversion of **10** was obviously retarded by the 4-*tert*-butyl group, but reliable rate constants could not be determined: **10** was rapidly destroyed at and above 3 °C through proton transfer from THF that generated the olefin **S6**. Moreover, **10** was soluble even in cyclopentane and could not be purified, so that the NMR signals of the coproducts overlapped those of **10** in samples that were prepared with *n*-BuLi both from the trimethylstannyl precursor **S2** (without evaporation) or directly from bromoalkene **S1** with evaporation. At lower temperatures, the $H_2C=$ group exhibited the expected AB-type ^{1}H NMR doublets with $^{2}J_{HH}$ = 8.6 Hz that characterizes trisolvation according to the Main Text.

In *t*-BuOMe as the solvent, $^{2}J_{HH}$ = 7.4 Hz indicated that **10** is a disolvated monomer like those listed in entries 5–8 of Table 1 in the Main Text. In Et_2O, **10** appeared to be a disolvated monomer with $^{2}J_{HH}$ = 7.0 Hz at 25 °C and possibly a certain trend toward trisolvation at −50 °C where $^{2}J_{HH}$ = 8.0 Hz.

Procedure. A dried NMR tube (5 mm) was charged with the bromoalkene [S1] **S1** (70 mg, 0.20 mmol) and anhydrous Et_2O (0.50 mL), then cooled at −50 °C under a stream of dry argon gas, and treated with *n*-BuLi (0.30 mmol) in hexanes (0.011 mL). After 10 min and subsequent warm-up, the solution contained the α-arylvinyllithium **10** and the lithium β-arylacetylide **S3** in a 4:1 ratio along with *n*-BuLi but very little olefin **S6** and no starting material **S1**. In futile attempts to obtain crystals of **10**, the opened NMR tube was placed in a big Schlenk tube that was fixed in an inclined position and evacuated cautiously under dry argon gas down to dryness (0.01 mbar). The foamy, yellow pot residue was dissolved under argon gas cover in one of the anhydrous solvents that were used for NMR measurements at variable temperatures.

A clear solution in cyclopentane displayed **10**, olefin **S6** (0.3 equiv), and Et_2O (1.2 equiv) that had remained coordinated at Li during the evaporation. Because this yellow solution deposited only a fine white powder (LiBr) in the course of four hours in a refrigerator, it was poured onto solid CO_2, warmed up, and dissolved in Et_2O (10 mL) and aqueous NaOH (2 M, 3 mL). The Et_2O layer was shaken with two further portions of NaOH (3 mL), then discarded. The combined aqueous phases were acidified and shaken with Et_2O (2 × 10 mL). The latter two Et_2O extracts were washed with dist. water until neutral, dried over Na_2SO_4, and evaporated to leave the acids **S4** [S1] and **S7** (64:36) as a white solid (48 mg, 76%).

^{1}H NMR of **10** (*t*-BuOMe, 80 MHz) δ 5.42 (d, ^{2}J = 7.4 Hz, 1H, β-H trans to aryl), 5.68 (d, ^{2}J = 7.4 Hz, 1H, β-H cis), 7.08 (s, 2H, 3-/5-H) ppm. ^{1}H NMR of **10** (Et_2O, 400 MHz, 25 °C) δ 1.302 (s, 9H, 4-CMe_3), 1.504 (s, 18H, 2-/6-CMe_3), 5.43 (d, ^{2}J = 7.0 Hz, Δδ = +0.06 ppm, 1H, β-H trans to aryl), 5.72 (d, ^{2}J = 7.0 Hz, Δδ = +0.79 ppm, 1H, β-H cis), 7.17 (s, 2 H, 3-/5-H) ppm. ^{1}H NMR of **10** (Et_2O, 400

MHz, –50 °C) δ 1.295, 1.517, 5.44 (d, ^2J = 8.0 Hz, 1H, β-H trans), 5.73 (d, ^2J = 8.0 Hz, 1H, β-H cis), 7.11 ppm.

2-(2´,4´,6´-Tri-*tert*-butylphenyl)hex-1-ene (S5).

This was obtained as a mixture with the olefin [S1] **S6**. ^{1}H NMR of **S5** ($CDCl_3$, 400 MHz) δ 0.94 (t, ^3J = 7.3 Hz, 3H, CH_3-6), 1.31 (s, 9H, 4´-CMe_3), 1.38 (m, ^3J = 7 Hz, 2H, CH_2-5), 1.41 (s, 18H, 2´-/6´-CMe_3), 1.57 (qi, ^3J = 7.5 Hz, 2H, CH_2-4), 2.26 (tdd, ^3J = 8 Hz, 2H, CH_2-3), 5.18 (dt, ^2J = 1.8 Hz, ^4J = 1.7 Hz, 1H, 1-H cis to aryl), 5.33 (dt, ^2J = 1.8 Hz, ^4J = 2.0 Hz, 1H, 1-H trans), 7.42 (s, 2H, 3´-/5´-H) ppm, assigned through comparison with **7** in the Main Text. ^{13}C NMR ($CDCl_3$, 100.6 MHz) δ 14.11 ($\mathit{C}H_3$-6), 22.60 ($\mathit{C}H_2$-5), 28.30 ($\mathit{C}H_2$-4), 31.44 (4´-C*Me*$_3$), 33.52 (2´-/6´-C*Me*$_3$), 34.78 (quart. 4´-C), 38.29 (quart. 2´-/6´-C), 40.95 ($\mathit{C}H_2$-3), 115.89 (C-1), 123.07 (C-3´/-5´), 139.98 (C-1´), 146.39 (C-2´/-6´), 147.18 (C-4´), 150.55 (C-2) ppm, assigned as above.

α-(2,4,6-Tri-*tert*-butylphenyl)acrylic acid (S7).

An acidic product mixture (56 mg) of **S4** and **S7**, obtained as described above from a cyclopentane solution of **10**, was recrystallized from hexane (15 mL) that deposited a felt of white needles (24 mg) which began to sublime at 230 °C and had a mp of 245–247 °C in a closed tubule.

^{1}H NMR ($CDCl_3$, 400 MHz) δ 1.32 (s, 9H, 4-CMe_3), 1.35 (s, 18H, 2-/6-CMe_3), 5.91 (d, ^2J = 1.71 Hz, 1H, β-H cis to aryl), 6.85 (d, ^2J = 1.71 Hz, 1H, β-H trans), 7.45 (s, 2H, 3-/5-H) ppm, assigned through selective {^{1}H} decoupling (see below). ^{13}C ($CDCl_3$, 100.6 MHz) δ 31.40 (q ^1J = 125.5 Hz, sept ^3J = 4.9 Hz, 4-C*Me*$_3$), 32.99 (q ^1J = 125.5 Hz, sept ^3J = 4.9 Hz, 2-/6-C*Me*$_3$), 34.96 (m, quart. 4-C), 37.72 (m, quart. 2-/6-C), 122.70 (dd, ^1J = 153.0 Hz, ^3J = 6.7 Hz, C-3/-5), 131.31 (m, C-1), 133.24 (sharp t, ^1J = 161.9 Hz, C-β), 142.40 [broadened d, ^2J = (–)3.6 Hz to trans-H only [S2], C-α], 147.99 (m, C-2/-6), 149.14 (m, C-4), 172.53 (dd, ^3J = 12.4 and 6.6 Hz, CO_2H) ppm, assigned through selective {^{1}H} decoupling as follows: {all C*H*$_3$} → C-2/-6 and C-4 as two s; {cis-H} → C-β as a d, C-1 simplified, CO_2H as a narrowed d; {trans-H} → C-β as a d, C-1 simplified, C-α as a s because of ^2J = 0 to cis-H [S2], CO_2H as a narrowed d; {3-/5-H} → C-1 as a dd. IR (KBr) ν 3500–2500 (broad H–O), 2963, 1696 (s), 1684 (s), 1613, 1362, 1196 cm^{-1}. Anal. Calcd for $C_{21}H_{32}O_2$ (316.48): C, 79.69; H, 10.19. Found: C, 79.54; H, 10.05.

2. References

S1. Knorr, R.; Rossmann, E. C.; Knittl, M.; Böhrer, P. *Tetrahedron* **2014**, *70*, 5332–5338.

S2. For negative and zero values of olefinic $^2J_{CH}$, see: Vögeli, U.; Herz, D.; von Philipsborn, W. *Org. Magn. Reson.* **1980**, *13*, 200–209, Table 1 therein.

3. Cis/trans diastereotopomerization rate constants

Table S1. Temperature-dependent pseudo-first-order rate constants k_ψ (s^{-1}) of monomeric α-(2,6-di-*tert*-butylphenyl)vinyllithium (**4**) in THF (47% by volume) and hydrocarbons at 400 MHz.

°C [a]	1000/T	conc.[b]	k_ψ	Δk_ψ	ln kψ	Δln kψ
–20	3.950	0.16	15.5	±0.5	2.74	±0.03
–12	3.829	0.10	24.0	±1.0	3.18	±0.04
–5	3.729	0.17	34.0	±2.0	3.53	±0.06
+1	3.648	0.09	46.0	±6.0	3.83	±0.13
+6	3.582	0.09	61.0	±9.0	4.11	±0.15
+10	3.532	0.16	78.0	±8.0	4.36	±0.10
+14	3.482	0.09	90.0	±10.0	4.50	±0.11
+25	3.354	0.13	150.0	±10.0	5.01	±0.07

[a] Uncertainty ±0.5 °C. [b] Concentrations of carbanion units in mol/L.

4. Tabulated primary NMR data

Table S2. Temperature dependence of the ^{1}H NMR chemical shifts δ of monomeric α-(2,6-di-*tert*-butylphenyl)vinyllithium (**4**&TMEDA) in THF (83% by volume) and hydrocarbons at 400 MHz. [a]

temp.	conc.	chemical shifts δ (ppm)							$^2J_{HH}$
°C	[**4**] [b]	3,5-H [c]	4-H [d]	cis-H [e]	trans-H [e]	*t*-Bu	CH_2N	NMe_2	Hz
+30	0.016	6.92	6.30	coal		1.43	2.31	2.15	–
+17	0.035	6.92	6.30	coal		1.42	2.30	2.15	–
–8	0.047	6.91	6.30	vbr	vbr	1.42	2.30	2.15	–
–50	0.048	6.90	6.29	5.59	5.29	1.42	2.29	2.14	8.5
–60	0.053	6.90	6.29	5.59	5.29	1.42	2.29	2.15	8.5
–75	0.057	6.89	6.29	5.58	5.29	1.42	2.29	2.15	8.5

[a] coal = in coalescence; vbr = very broad. [b] Concentrations of carbanion units in mol/L.
[c] Doublet ^3J = 7.8 Hz. [d] Triplet ^3J = 7.8 Hz [e] In relation to α-aryl.

Table S3. Temperature dependence of the ^{1}H NMR chemical shifts δ of monomeric α-(2,6-di-*tert*-butylphenyl)vinyllithium (**4**) in THF (47% by volume), with *n*-$BuSnMe_3$, hydrocarbons, and MeLi at 400 MHz. [a]

temp.	conc.	chemical shifts δ (ppm)					$^2J_{HH}$
°C	[**4**] [b]	3,5-H [c]	4-H [d]	cis-H [e]	trans-H [e]	*t*-Bu	Hz
+34	0.04	6.94	6.32	xbr	xbr	1.43	–
+25	0.13	6.93	6.31	(5.59) vbr	(5.34) vbr	1.42	–
+23 [f]	0.10	6.93	6.31	coal		–	–
+20	0.08	6.93	6.32	(5.61) br	(5.32) br	1.43	–
+14	0.09	6.93	6.32	(5.62) br	(5.32) br	1.43	–
+10	0.16	6.92	6.31	(5.61) br	(5.31) br	1.43	–
+6	0.09	6.92	6.31	5.62 br	5.31 br	1.43	–
+1	0.09	6.92	6.31	5.62 br	5.31 br	1.43	–
–5	0.17	6.92	6.31	5.62	5.31	1.43	brd
–12	0.10	6.92	6.31	5.62	5.31	1.43	brd
–20	0.16	6.91	6.30	5.61	5.30	1.43	brd
–45	0.16	6.91	6.30	5.60	5.29	1.43	8.5
–85	0.17	6.90	6.30	5.59	5.28	1.43	8.5
–100	0.18	6.90	6.30	5.58	5.28	1.43	brd

[a] coal = in coalescence; br = broad; brd = broadened; vbr = very broad; xbr = extremely broad. [b] Concentrations of carbanion units in mol/L. [c] Doublet ^3J = 7.8 Hz. [d] Triplet ^3J = 7.8 Hz. [e] In relation to α-aryl. [f] At 200 MHz.

Table S4. Temperature dependence of the ^{1}H NMR chemical shifts δ of monomeric α-(2,4,6-tri-*tert*-butylphenyl)vinyllithium (**10**) in THF at 400 MHz.

temp.	chemical shifts δ (ppm)					$^2J_{HH}$
°C	3,5-H [a]	cis-H	trans-H	2,6-*t*-Bu_2	4-*t*-Bu	Hz
–60	7.19	5.65	5.31	1.50	1.26	8.6
–82	7.18	5.64	5.30	1.50	1.26	8.6

[a] Singlet.

Table S5. Temperature dependence of the ^{1}H NMR chemical shifts δ of monomeric α-(2,6-di-*tert*-butylphenyl)vinyllithium (**4**&TMEDA) in *t*-BuOMe (77% by volume) and cyclopentane (22%) at 400 MHz. [a]

temp.	conc.	chemical shifts δ (ppm)							conc. (M)	$^2J_{HH}$
°C	[**4**] [b]	3,5-H [c]	4-H [d]	cis-H [e]	trans-H [e]	*t*-Bu	CH_2N	NMe_2	TMEDA	Hz
+25	0.055	6.94	6.34	5.63	5.29	1.44	2.35	2.18	0.072	7.4
–24	0.058	6.93	6.34	5.62	5.29	1.44	2.36	2.18	0.082	7.4
–68	0.057	6.93	6.34	5.61	5.28	1.44	2.38 vbr	2.18 brd	0.070	7.4
–88	0.057	6.93	6.35	5.59	5.27	1.44	ca. 2.3 [f]<br>2.41 vbr[g]	2.14 [f]<br>2.20 [g]	0.015 [f]<br>0.060 [g]	7.4<br>7.4

[a] brd = broadened; vbr = very broad. [b] Concentrations of carbanion units in mol/L. [c] Doublet ^3J = 7.8 Hz. [d] Triplet ^3J = 7.8 Hz. [e] In relation to α-aryl. [f] Free. [g] Coordinated.

Table S6. Temperature dependence of the ^{1}H NMR chemical shifts δ of monomeric α-(2,6-di-*tert*-butylphenyl)vinyllithium (**4**&TMEDA) in [D_8]toluene (85% by volume) at 400 MHz. [a]

temp.	conc.	chemical shifts δ (ppm)							conc. (M)	$^2J_{HH}$
°C	[**4**] [b]	3,5-H [c]	4-H [d]	cis [e]	trans [e]	*t*-Bu	CH_2N	NMe_2	TMEDA	Hz
+60	0.025	7.28	6.70	6.06 br	5.49 br	1.65	1.88	1.93	0.130	br
+45	0.079	7.30	6.72	6.09	5.51	1.67	1.84	1.90	0.170	brd
+25	0.126	7.32	6.74	6.12	5.52	1.69	1.77 br	1.84	0.176	7.3
–20	0.089	7.36	6.80	6.20	5.58	1.71	xbr	xbr	(0.136)	7.3
–44	0.083	7.40	6.84	6.26	5.62	1.73	xbr [f]<br>–	xbr [f]<br>(1.58) [g]	0.055 [f]<br>0.097 [g]	7.3<br>br
–56	0.127	7.41	6.85	6.26	5.63	1.74	xbr [f]<br>–	vbr [f]<br>(1.57) [g]	0.039 [f]<br>–	7.4<br>shp
–68	0.125	7.43	6.87	6.29	5.65	1.75	(2.3) [f]<br>–	(2.1) [f]<br>(1.53) [g]	0.037 [f]<br>–	7.4<br>shp
–82	0.022	7.47	ca. 6.9	6.38	5.70	1.78	2.30 [f]<br>–	2.07 [f]<br>–	0.039 [f]<br>–	7.4<br>brd

[a] br = broad; brd = broadened; vbr = very broad; xbr = extremely broad; shp = sharp. [b] Concentrations of carbanion units in mol/L. [c] Doublet ^3J = 7.8 Hz. [d] Triplet ^3J = 7.8 Hz. [e] In relation to α-aryl. [f] Free. [g] Coordinated.

Table S7. Temperature dependence of the ^{1}H NMR chemical shifts δ of monomeric α-(2,6-di-*tert*-butylphenyl)vinyllithium (**4**) in TMEDA (64% by volume) with hydrocarbons and MeLi at 400 MHz. [a]

temp. °C	conc. [**4**] [b]	chemical shifts δ (ppm) 3,5-H [c]	4-H [d]	cis-H [e]	trans-H [e]	*t*-Bu	$^2J_{HH}$ Hz
+23 [f]	0.077	6.93	6.34	5.64 br	5.28 br	1.44	br
+25	0.030	6.95	6.35	5.66	5.28	1.44	ca. 7.4
+25	0.018	6.94	6.35	5.65	5.28	1.44	ca. 7.4
+25	0.010	6.95	6.36	5.66	5.29	1.44	ca. 7.4
+10	0.020	6.93	6.35	5.65	5.28	1.44	7.4
–22	0.012	6.92	6.33	5.63	5.28	(1.44)	7.4

[a] br = broad. [b] Concentrations of carbanion units in mol/L. [c] Doublet ^3J = 7.8 Hz. [d] Triplet ^3J = 7.8 Hz. [e] In relation to α-aryl. [f] At 200 MHz.

Table S8. Temperature dependence of the ^{1}H NMR chemical shifts δ of monomeric α-(2,6-di-*tert*-butylphenyl)vinyllithium (**4**) in Et_2O (54% by volume) with cyclopentane, *n*-BuLi and MeLi at 400 MHz. [a]

temp. °C	conc. [**4**] [b]	chemical shifts δ (ppm) 3,5-H [c]	4-H [d]	cis-H [e]	trans-H [e]	*t*-Bu	$^2J_{HH}$ Hz
+36	0.14	7.00	6.41	5.64 brd	5.34 brd	1.44	brd
+23 [f]	0.15	7.00	6.40	5.63	5.34	1.44	7.4
+25	0.10	7.01	6.42	5.64	5.34	1.44	7.4
+25	0.11	7.00	6.41	5.64	5.34	1.44	7.4
+25	0.15	7.00	6.40	5.63	5.34	1.44	7.4
+25 [g]	0.018	6.95	6.34	5.64	5.31	1.44	7.4 shp
+11 [g]	0.017	6.95	6.34	5.64	5.30	1.44	7.4
–30	0.09	6.96 br	6.37 br	5.62	5.35	1.44	7.4
–66	0.12	6.96	6.35	5.61	5.35	1.44	7.4
–83	0.09	6.96	6.35	5.61 brd	5.35 brd	1.44 br	7.4
–85	0.14	6.95	6.35	5.60 brd	5.35 brd	1.44 brd	(7.5)

[a] br = broad; brd = broadened; shp = sharp. [b] Concentrations of carbanion units in mol/L. [c] Doublet ^3J = 7.8 Hz. [d] Triplet ^3J = 7.8 Hz. [e] In relation to α-aryl. [f] At 200 MHz. [g] In Et_2O (80%) with *free* TMEDA.

Table S9. Temperature dependence of the ^{1}H NMR chemical shifts δ of aggregated α-(2,6-di-*tert*-butylphenyl)vinyllithium in [D_8]toluene with Et_2O (ca. 2 equiv) and *n*-BuLi at 400 MHz. [a]

temp.	conc.	chemical shifts δ (ppm)							Et_2O	$^{2}J_{HH}$
°C	[**4**] [b]	3,5-H [c]	4-H [d]	cis-H [e]	trans-H [e]	*t*-Bu	Et_2O	Et_2O	(M)	Hz
+75	0.03	7.22	6.66	xbr		1.49	3.24	1.02	0.26	–
+55	0.09	7.27	6.78	(5.56) xbr		1.48	3.23	1.00	0.25	–
+25	0.12	7.32	6.83	5.91 vbr	5.24 br	1.53	3.19 brd	0.97	0.25	–
+23 [f]	–	7.30	6.81	5.86 vbr	5.25 vbr	1.51	3.30 brd	0.94	–	–
–55	0.07	7.41	6.94	5.97	5.30	1.64	2.99 br	0.95	0.25	3.7 brd
–72	0.08	7.42	obsc	6.00	5.32	1.66	2.97 br	0.96	0.23	brd
–84	0.08	7.44	6.98	6.03	5.33	1.67	(3.04) [g]<br>–	0.98 [g]<br>0.61 br [h]	0.23<br>–	brd

[a] br = broad; brd = broadened; vbr = very broad; xbr = extremely broad; obsc = obscured. [b] Concentrations of carbanion units in mol/L. [c] Doublet ^{3}J = 7.8 Hz. [d] Triplet ^{3}J = 7.8 Hz. [e] In relation to α-aryl. [f] At 200 MHz. [g] Free. [h] Coordinated.

Table S10. Temperature dependence of the ^{13}C NMR chemical shifts δ of monomeric α-(2,6-di-*tert*-butylphenyl)vinyllithium (**4**&TMEDA) in THF (83% by volume) and hydrocarbons at 100.6 MHz. [a]

temp.	conc.	chemical shifts δ (ppm)									
°C	[**4**] [b]	C-α	C-1	C-2,6	C-3,5	C-4	C-*β*	2,6-C	C*Me*$_3$	CH_2N	NCH_3
–8	0.047	214.9 shp	163.7	138.3	123.4	116.5	112.2	37.6	32.2	58.8	46.2
–50	0.048	214.8 (t)	163.7	138.1	123.3	116.4	112.1	37.5	32.0	58.7	46.3
–60	0.053	214.8 t [c]	163.7	136.1	123.3	116.4	112.1	37.5	31.9	58.7	46.3
–75	0.057	214.8 t [c]	163.7	138.0	123.3	116.3	112.1	37.6	31.9	58.7	46.3

[a] shp = sharp; t = triplet. [b] Concentrations of carbanion units in mol/L. [c] $^{1}J_{C,Li}$ = 10.8 Hz.

Table S11. Temperature dependence of the ^{13}C NMR chemical shifts δ of monomeric α-(2,6-di-*tert*-butylphenyl)vinyllithium (**4**) in THF (47% by volume), with *n*-BuSnMe$_3$, hydrocarbons, and MeLi at 100.6 MHz. [a]

temp.	conc.	chemical shifts δ (ppm)							
°C	[**4**] [b]	C-α	C-1	C-2,6	C-3,5	C-4	C-*β*	2,6-C	C*Me*$_3$
+25	0.13	214.7	163.5	138.2	123.4	116.5	112.2	37.5	32.1
–20	0.16	214.5	163.5	138.2	123.4	116.5	112.2	37.5	32.1
–45	0.16	214.6 br	163.6	138.1	123.3	116.5	112.2	37.5	32.0
–85	0.17	214.6 t [c]	163.6	138.0	123.3	116.4	112.1	37.5	31.8
–100	0.18	214.6 t [c]	163.6	137.9	123.3	116.3	112.0	37.5	31.7

[a] t = triplet. [b] Concentrations of carbanion units in mol/L. [c] $^1J_{C,Li}$ = 10.8 Hz.

Table S12. Temperature dependence of the ^{13}C NMR chemical shifts δ of monomeric α-(2,4,6-tri-*tert*-butylphenyl)vinyllithium (**10**) in THF at 100.6 MHz.

temp.	chemical shifts δ (ppm)									
°C	C-α	C-1	C-2,6	C-3,5	C-4	C-*β*	2,6-C	4-C	2,6-C*Me*$_3$	4-C*Me*$_3$
+3	–	160.6	137.8	120.2	136.8	112.5	38.0	37.3	32.4	30.2
–60	(215.3)	161.0	137.8	120.2	136.4	112.5	37.9	37.3	32.1	30.5
–82	215.3 t [a]	161.0	137.7	120.1	136.3	112.5	37.9	37.3	32.0	30.4

[a] t = triplet, $^1J_{C,Li}$ = 10.9 Hz.

Table S13. Temperature dependence of the ^{13}C NMR chemical shifts δ of monomeric α-(2,6-di-*tert*-butylphenyl)vinyllithium (**4**&TMEDA) in *t*-BuOMe (77% by volume) and cyclopentane (22%) at 100.6 MHz. [a]

temp.	conc.	chemical shifts δ (ppm)									
°C	[**4**] [b]	C-α	C-1	C-2,6	C-3,5	C-4	C-*β*	2,6-C	C*Me*$_3$	CH_2N	NCH_3
+25	0.055	212.1	161.2	137.6	123.8	117.1	111.9	37.7	32.4	57.8	45.9
–24	0.058	211.9 (t)	161.2	137.2	123.8	117.0	111.7	37.6	32.1	57.5 brd	45.9
–68	0.057	211.9 t [c]	161.1	137.1	123.8	116.9	111.6	37.6	31.9	58.7 brd[d]<br>56.8 [e]	46.3 brd[d]<br>45.7 [e]
–88	0.057	211.8 t [c]	161.2	137.0	123.8	116.8	111.5	37.7	31.8	58.7 [d]<br>56.7 [e]	46.4 [d]<br>45.7 [e]

[a] brd = broadened; t = triplet. [b] Concentrations of carbanion units in mol/L. [c] $^1J_{C,Li}$ = 13.9 Hz. [d] Free. [e] TMEDA (1 equiv) coordinated.

Table S14. Temperature dependence of the ^{13}C NMR chemical shifts δ of monomeric α-(2,6-di-*tert*-butylphenyl)vinyllithium (**4**&TMEDA) in [D_8]toluene (85% by volume) at 100.6 MHz. [a]

temp.	conc.	chemical shifts δ (ppm)									
°C	[**4**] [b]	C-α	C-1	C-2,6	C-3,5	C-4	C-*β*	2,6-C	C*Me*$_3$	CH_2N	NCH_3
+25	0.126	212.4	161.1	137.7	124.1	117.3	111.8	37.7	32.4	56.9	45.4
–20	0.089	212.2 vbr	160.9	137.2	124.1	117.2	111.6	37.7	32.1	56.6 vbr	45.3 vbr
–44	0.083	212.1 (t)	160.8	137.0	124.1	117.1	111.5	37.7	31.9	57.7 xbr [d]<br>55.8 br [e]	46.1 br [d]<br>44.8 br [e]
–56	0.127	212.0 t [c]	160.9	137.0	124.1	117.1	111.4	37.7	31.9	(57) [d]<br>55.6 [e]	46.0 br [d]<br>44.8 brd [e]
–68	0.125	212.0 t [c]	160.9	137.0	124.1	117.0	111.3	37.7	31.8	–<br>55.5 [e]	46.2 br [d]<br>44.6 br [e]
–82	0.022 [g]	–	160.8	136.8	124.2	117.0	111.3	37.7	31.7	58.3 [d]<br>55.3 [f]	46.2 shp [d]<br>xbr [f]

[a] br = broad; brd = broadened; vbr = very broad; xbr = extremely broad; shp = sharp; t = triplet. [b] Concentrations of carbanion units in mol/L. [c] $^{1}J_{C,Li}$ = 13.8 Hz. [d] Free. [e] TMEDA (1 equiv) coordinated. [f] Coordinated. [g] Weakly soluble.

Table S15. Temperature dependence of the ^{13}C NMR chemical shifts δ of monomeric α-(2,6-di-*tert*-butylphenyl)vinyllithium (**4**) in TMEDA (64% by volume) with hydrocarbons and MeLi at 100.6 MHz. [a]

temp.	conc.	chemical shifts δ (ppm)							
°C	[**4**] [b]	C-α	C-1	C-2,6	C-3,5	C-4	C-*β*	2,6-C	C*Me*$_3$
+25	0.030	211.8 brd	160.7	137.3	123.8	117.2	111.9	37.7	32.4
+25	0.010	–	160.8	137.3	123.8	117.2	111.9	37.7	32.4
+10	0.020	–	160.9	137.2	123.8	117.1	111.9	37.7	32.4
–22	0.012	–	161.2	137.1	123.7	116.9	111.8	37.6	32.3

[a] brd = broadened. [b] Concentrations of carbanion units in mol/L.

Table S16. Temperature dependence of the ^{13}C NMR chemical shifts δ of monomeric α-(2,6-di-*tert*-butylphenyl)vinyllithium (**4**) in Et_2O (54% by volume) with cyclopentane, *n*-BuLi and MeLi at 100.6 MHz. [a]

temp.	conc.	chemical shifts δ (ppm)							
°C	[**4**] [b]	C-α	C-1	C-2,6	C-3,5	C-4	C-β	2,6-C	C*Me*$_3$
+25	0.10	209.3	161.6	138.8	124.2	118.1	113.3	37.7	32.3
+25	0.15	209.6	161.8	138.7	124.1	118.0	113.2	37.7	32.3
+11 [c]	0.017	– [d]	– [d]	137.6	123.9	117.1	112.0	37.7	32.2
–10 [c]	–	– [d]	– [d]	137.4	123.9	117.0	112.0	37.7	32.1
–30	0.09	210.3	162.0	137.8	124.0	117.3	112.6	37.7	32.0
–66	0.12	210.3 br	162.0	137.5	123.9	117.1	112.4	37.7	31.8
–83	0.09	210.2 t [e]	162.0	137.4	123.9	117.1	112.2	37.7	31.7
–85	0.14	210.3 t [e]	162.0	137.3	123.9	117.0	112.2	37.7	31.7

[a] br = broad; t = triplet. [b] Concentrations of carbanion units in mol/L. [c] In Et_2O (80%) with *free* TMEDA. [d] Weakly soluble. [e] $^1J_{C,Li}$ = 13.7 Hz.

Table S17. Temperature dependence of the ^{13}C NMR chemical shifts δ of aggregated α-(2,6-di-*tert*-butylphenyl)vinyllithium in [D_8]toluene with Et_2O (ca. 2 equiv) and *n*-BuLi at 100.6 MHz. [a]

temp.	conc.	chemical shifts δ (ppm)									
°C	[**4**] [b]	C-α	C-1	C-2,6	C-3,5	C-4	C-β	2,6-C	C*Me*$_3$	Et_2O	Et_2O
+55	0.09	–	–	140.9 vbr	125.3	120.8 br	115.4 br	37.1	32.5	65.6	15.2
+25	0.12	–	–	141.0 br	125.1	120.9 br	115.5 br	37.4	32.3	65.0	14.9
–55	0.07	199.1	159.7	141.4 shp	125.0	120.8	115.4	37.4	31.9	63.6 vbr	14.6 xbr
–72	0.08	198.7 br	159.6	141.2 shp	125.0	120.7	115.3	37.4	31.8	(64.1) xbr	14.6 xbr
–84	0.08	198.6 br	159.6	141.3 shp	125.0	120.7	115.4	37.4	31.8	65.1 xbr	14.6 xbr

[a] br = broad; vbr = very broad; xbr = extremely broad; shp = sharp. [b] Concentrations of carbanion units in mol/L.
